# Prognosis prediction based on methionine metabolism genes signature in gliomas

**DOI:** 10.1186/s12920-023-01754-x

**Published:** 2023-12-06

**Authors:** Sujin Zhou, Xianan Zhao, Shiwei Zhang, Xue Tian, Xuepeng Wang, Yunping Mu, Fanghong Li, Allan Z. Zhao, Zhenggang Zhao

**Affiliations:** School of Biomedical and Pharmaceutical Sciences, Guangdong University of Technology, 510006 Guangzhou, Guangdong Province China

**Keywords:** Methionine metabolism, Glioma, Prognostic model, Overall survival, Gene signature

## Abstract

**Background:**

Glioma cells have increased intake and metabolism of methionine, which can be monitored with 11 C-L-methionine. However, a short half-life of 11 C (~ 20 min) limits its application in clinical practice. It is necessary to develop a methionine metabolism genes-based prediction model for a more convenient prediction of glioma survival.

**Methods:**

We evaluated the patterns of 29 methionine metabolism genes in glioma from the Cancer Genome Atlas (TCGA). A risk model was established using Lasso regression analysis and Cox regression. The reliability of the prognostic model was validated in derivation and validation cohorts (Chinese Glioma Genome Atlas; CGGA). GO, KEGG, GSEA and ESTIMATE analyses were performed for biological functions and immune characterization.

**Results:**

Our results showed that a majority of the methionine metabolism genes (25 genes) were involved in the overall survival of glioma (logrank p and Cox p < 0.05). A 7-methionine metabolism prognostic signature was significantly related to a poor clinical prognosis and overall survival of glioma patients (C-index = 0.83). Functional analysis revealed that the risk model was correlated with immune responses and with epithelial-mesenchymal transition. Furthermore, the nomogram integrating the signature of methionine metabolism genes manifested a strong prognostic ability in the training and validation groups.

**Conclusions:**

The current model had the potential to improve the understanding of methionine metabolism in gliomas and contributed to the development of precise treatment for glioma patients, showing a promising application in clinical practice.

**Supplementary Information:**

The online version contains supplementary material available at 10.1186/s12920-023-01754-x.

## Background

Gliomas are one of the most lethal and frequent brain tumors [1], with an estimated 100,000 new cases being diagnosed annually [2]. Based on the fifth edition of the World Health Organization (WHO) classification of the central nervous system tumors [3], histological classification and molecular biomarkers, such as grade I to IV, isocitrate dehydrogenase 1 and 2 (IDH1/IDH2)mutation and chromosome 1p/19q co-deletion status, have been used in diagnosis, treatment and prognostic evaluation of glioma [4]. However, both histological and molecular approach are less accurate on an individual basis. Furthermore, therapies targeting these molecular markers have a limited overall benefit. There is an urgent need for discovering additional prognostic biomarkers beyond the WHO classification to better inform prognosis prediction and treatment strategy.

Glioma cells have increased methionine (Met) consumption and metabolism, which can be measured with 11 C-L-methionine. Several studies demonstrated that the 11 C-methionine PET scan is highly sensitive and specific for detecting high-grade gliomas [5, 6]. Furthermore, lower Met absorption or a decrease in the Met metabolic tumor volume was associated with a better long-term prognosis, indicating that 11 C-methionine PET can be used as a prognostic indicator for glioma patients [7]. Although there is abundance of data available about the 11 C-methionine PET for assessing, identifying, and grading gliomas [8], no study about the prognostic value of methionine metabolism genes in glioma patients has been reported.

This study investigated the methionine metabolism genes in gliomas applying bioinformatics techniques [9–12]. A prediction signature based on 7-methionine metabolism genes was developed. Next, functional enrichment analysis was performed to investigate the underlying pathways. Additionally, we developed a nomogram combining clinic pathological characteristics with the Met metabolism prognostic signature, and discovered that it performed exceptionally well in assessing the 1-, 2-, 3-, 5- and 10- year survival rates of glioma patients. The currently developed system could improve our understanding of methionine metabolism in gliomas, allowing us to provide more precise and effective treatment for glioma patients.

## Methods

### Data source and ethics approval

The RNA-seq data comprising a merged cohort of low-grade glioma (LGG) and GBM containing 691 patients/samples were obtained from TCGA databases (https://tcgadata.nci.nih.gov) [13]. IDH mutation, 1p/19q, transcriptional subtyping, and MGMT promoter methylation information were obtained from the supplementary data from a previous publication [14]. The CGGA mRNA expression data (mRNAseq_325, mRNAseq_693 and mRNA-array) and corresponding clinicopathological features were collected from the CGGA database (https://www.cgga.org.cn) [15].

Single-cell sequencing data were collected from the GSE117891 [16] data set of the Gene Expression Omnibus database. As the current study was an analysis based on publicly available databases with pre-existing institutional review board (IRB) approval, ethical statement and informed consent was therefore waived.

### Assembling a set of methionine metabolism genes

Genes related to methionine metabolism were downloaded from the gene sets, including the WP_CYSTEINE_AND_METHIONINE_CATABOLISM and WP_METHIONINE_DE_NOVO_AND_SALVAGE_PATHWAY gene sets, in the Molecular Signatures Database (MSigDB) version 2023.1. Hs. Methionine metabolism gene set containing 29 genes was finally obtained after removing overlapping genes and four low-expressed genes showing an undetectable level of expression in more than 50% of samples in TCGA and CGGA database (BHMT, MAT1A, TAT, and SQOR).

### Survival analysis

Survival distribution and significance was evaluated by Kaplan-Meier curves, log-rank test analysis, univariate Cox regression and multivariate Cox model analyses with the survival and survminer packages in R software (version 4.2.2, http://www.r-project.org.The R package survivalROC was applied to establish time-dependent receiver operating characteristic (ROC) curve to assess the accuracy of the risk signatures in predicting the outcomes for glioma patients. A risk plot was drawn using Pheatmap R package to show the distribution of survival status of samples in different risk groups.

### Single-cell RNA-seq analysis

The processed scRNAseq UMI Count Matrix and metadata were obtained from GSE117891. The Seurat package was used to perform cell clustering (version 4.1.0). Standard preprocessing workflow of single-cell sequencing results was conducted according Seurat - Guided Clustering Tutorial. CD68, FA2H, CD3E, and SOX2 are markers for macrophages, oligodendrocytes, T lymphocytes, and tumor cells, respectively [16]. The scRNAseq expression data visualization was realized using ggplot2 packages in R software.

### Methionine metabolism gene signature construction and validation

The Methionine metabolism genes related to overall survival (logrank p and Cox *p* < 0.001) were analyzed by least absolute shrinkage and selection operator (LASSO) regression using the glmnet R package in TCGA database to narrow the range of prognosis-related genes. Subsequently, the Akaike information criterion (AIC) method of multivariate Cox regression analysis was conducted using the MASS package to build an optimal methionine metabolism gene risk signature based on the results of multivariate Cox regression analysis. The risk score was calculated using the formula: Riskscore=∑_Ni=1_(Coefi×Expi),where Expi is the expression value of the methionine metabolism genes and Coefi is the corresponding regression coefficient calculated by multivariate Cox regression analysis. TCGA data served as the training cohort, whereas CGGA325, CGGA mRNA array and CGGA693 data were as the validation cohorts.

### Differentially expressed gene (DEG) and functional enrichment analysis

The mRNA count data from CGGA-325 database was used as the input data, and DEG analysis was conducted using the DEGseq R package. The Principal Component Analysis (PCA) was applied to visualize variation between samples in DESeq2 package. The definition of DEGs was genes with false discovery rate (FDR) value < 0.05 and Log2 (fold change (FC)) > 1. The Gene Ontology (GO), Kyoto Encyclopedia of Genes and Genomes (KEGG) and hallmark gene sets were used to annotate biological pathways employing the clusterProfiler R package [17].

### Mutation and drug susceptibility analysis

The mutation annotation format (MAF) from the TCGA database was generated using R package “maftools”, and the somatic mutations of gliomas in low- and high-risk groups were plotted. The total number of missense, insertions/deletions, and frameshift variations found in the tumor sample were added, and the tumor mutation burden (TMB) of each glioma’s patient in the TCGA cohort was also calculated. Drug sensitivity analysis was performed using R package “pRRophetic” [18].

### ESTIMATE analysis

The immunological landscape was assessed by performing ESTIMATE analysis. The ESTIMATE R package examined the computation of ESTIMATE scores, immunological scores, or stromal scores [19].

### Development and evaluation of a nomogram based on the gene signature

A nomogram was developed using the “rms” R package to facilitate the prediction of 1-, 2-, 3-, 5- and 10- year overall survival (OS) chance in glioma patients. Calibrate curves and C-Index values were further used to show the accuracy of the survival prediction from the nomogram.

### Statistical analysis

Unpaired Student’s t test was used to determine the statistical significance for variables with two normally distributed comparison groups. Wilcoxon test was used to calculate the statistical significance between the two groups for variables with nonnormal distributions. One-way ANOVA test was applied as parametric methods to compare more than two groups, whereas Kruskal-Wallis test was employed as nonparametric approaches. Between two groups, linear relationship determination using Pearson’s or Spearman’s correlation analysis was applied. For all statistical studies, R and SPSS software was used. *P* < 0.05 was considered as statistically significant.

## Results

### Methionine metabolism gene expression is associated with clinical outcomes of gliomas

**Fig. 1:**
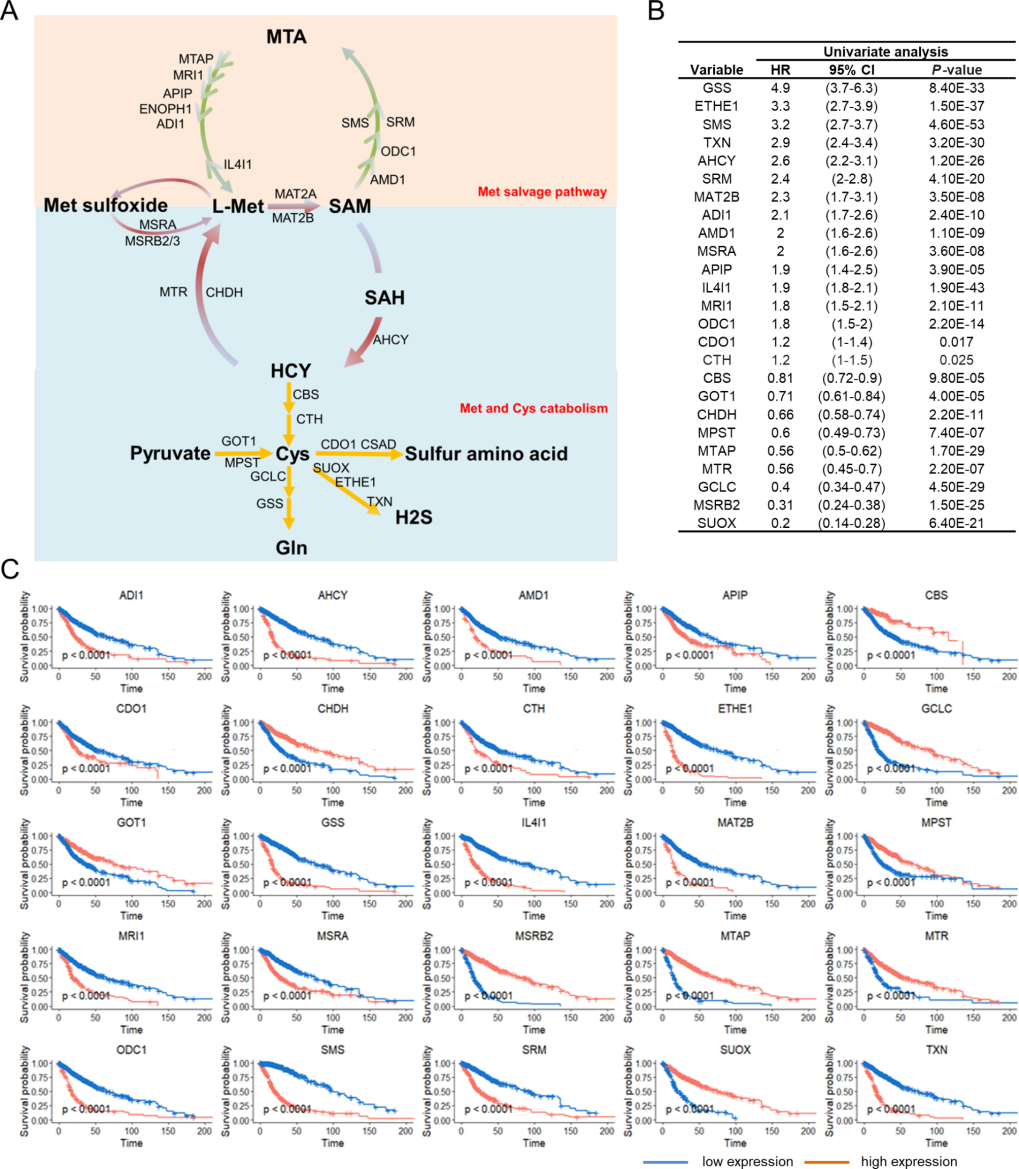
**Met metabolism genes expression is associated with clinical outcomes of gliomas. (A)** Schematic of methionine metabolism pathways. MTA, 5′-methylthioadenosine; L-Met, L-methionine; SAM, S-adenosylmethionine; SAH, S-Adenosylhomocysteine; HCY, Homocysteine; Cys, Cysteine; Gln, Glutamine. **(B, C)** Clinical outcomes of glioma patients with low and high expression of methionine metabolism genes. Univariate clinical prognostic parameter analysis in TCGA databases. Abbreviations: CI, confidence interval; HR, hazard ratio **(B)**; Kaplan-Meier survival analysis was performed in TCGA databases between low (blue) and high (red) expression levels **(C).** Survival differences were compared based on the division of the expression of methionine metabolism genes across the median into high and low

To understand the clinical significance of Met metabolism genes, a total of 29 genes, including 10 genes involved in methionine de novo and salvage pathways and 19 genes related to methionine and cysteine catabolism (Fig. 1A), were identified from MSigDB. The prognostic value of these genes in the TCGA dataset was investigated using Kaplan-Meier and Cox proportional hazard model analysis. After splitting patients into high and low groups down the median, 25 genes were found to be significantly associated to glioma patients’ overall survival (logrank p and Cox *p* < 0.05, Fig. 1B, C). We then investigated the expression distribution of these genes using a single-cell RNA transcriptome (GSE117891). Dotplot revealed that most Met metabolism genes were generally expressed after identifying cell types by the known unique signature genes (Fig. S1A-E). These results indicated the potential role of Met metabolism genes in gliomas prognosis.

### Establishment of a Met metabolism genes signature for gliomas

**Fig. 2:**
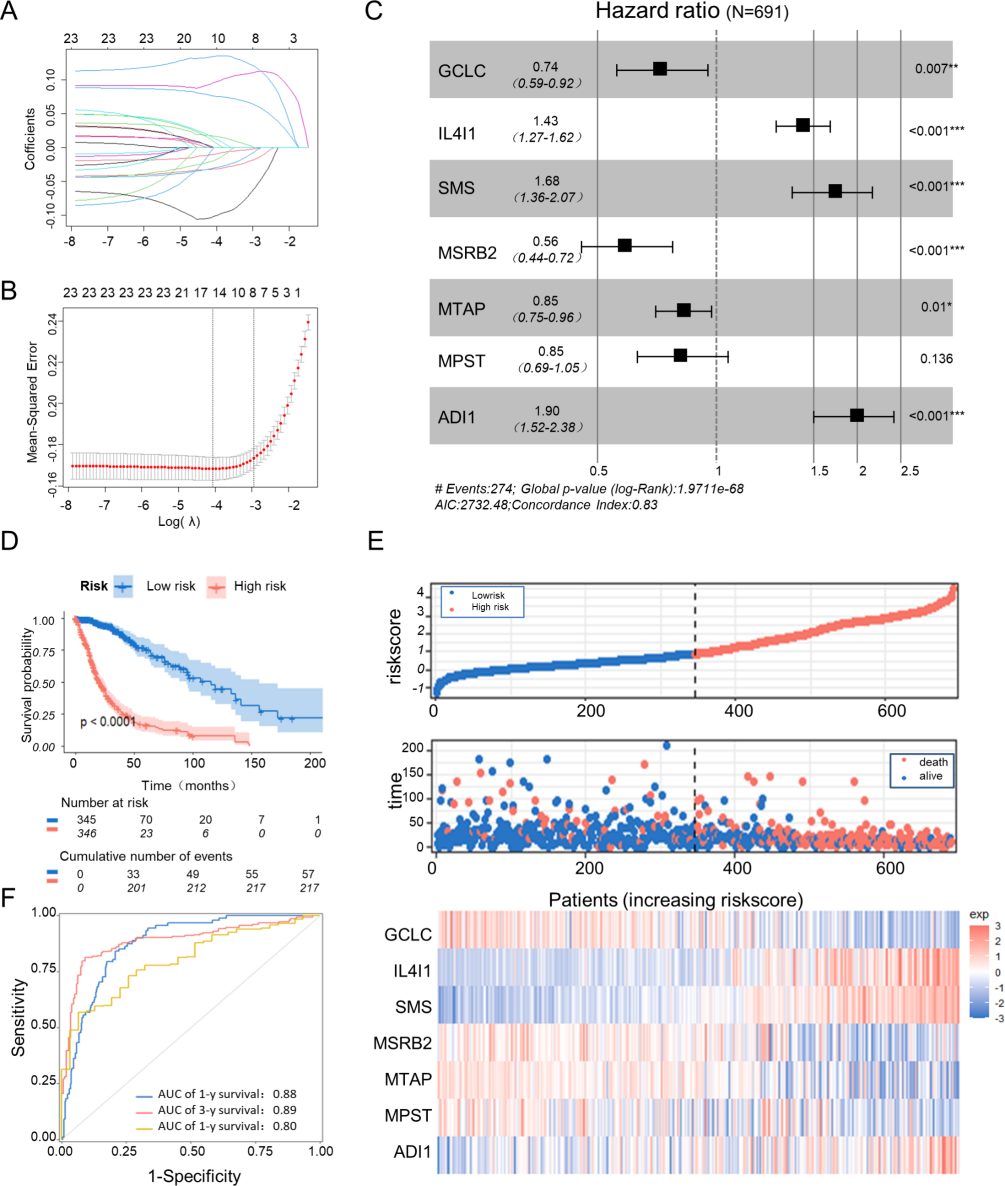
**The development and evaluation of a prognostic model of the Met metabolism gene risk signature for gliomas. (A**) LASSO coefficient profiles of the 23 genes in the TCGA data set. **(B)** Selection of the optimal parameter (lambda) in the Lasso model. **(C)** Seven genes were chosen for establishing a prognosis signature. **(D)** Kaplan–Meier survival analysis of high-risk groups (red) and low-risk groups (blue). **(E)** Analysis of risk score, survival status, and survival time between two risk groups, as well as the expression distribution of the Seven Met metabolism gene signature in the TCGA dataset. **(F)** Time-dependent ROC curves demonstrated the AUC value of prognostic model

To develop a convenient prediction model for gliomas, a set of 23 Met metabolism-related genes (logrank p and Cox *p* < 0.001, Fig. 1B, C) were subjected to the LASSO regression analysis (Fig. 2A, B). The Akaike’s Information Criterion method of multivariate Cox regression analysis was performed on the genes returned from the Lasso regression analysis (12 genes) to develop an optimal model (Concordance index, C-index = 0.83, Fig. 2C), which included seven genes, namely, GCLC, IL4I1, SMS, MSRB2, MTAP, MPST, and ADI1 with a cox regression coefficient of -0.3024212, 0.3596390, 0.5178925, -0.5721255, -0.1594767, -0.1611646 and 0.6415830, respectively. Then, the survival risk score for each patient in the TCGA dataset was calculated based on the expression level of the seven candidate genes and their related coefficients. According to the median risk score, the patients were classified into high- and low-risk groups, with the high-risk group showing significantly lower survival rates than the low-risk group (*P* < 0.0001, Fig. 2D, E). A remarkably lower expression was in GCLC, MSRB2, MTAP and MPST in the high-risk groups was noted, whereas IL4I1, SMS, and ADI1 expression was significantly higher (Fig. 2E). The protein expression levels of the seven genes were also collected from the Human Protein Atlas (HPA) (https://www.proteinatlas.org/). The expression levels of risk factors IL4I1, SMS, and ADI1 in high-grade gliomas were higher than those in lower‐grade gliomas, while the protein expression levels of protective factor GCLC, MSRB2, MTAP and MPST were exactly the opposite (Fig. S2).

Time-dependent ROC curves were used to assess the sensitivity and specificity of the prognostic prediction by the Met metabolism risk signature. The area under the ROC curves (AUCs) for predicting 1-, 3-, and 5-year survival times were 0.88, 0.89, and 0.80, respectively (Fig. 2F). IDH wild-type (IDH wt), IDH mutant samples with codeletion of chromosomal arms 1p19q (IDH mutant-codel) and samples with euploid 1p/19q (IDH mutant-non-codel) have been proposed as classifications for glioma regardless of grade and histology. The prognostic value of Met metabolism risk signature in the TCGA dataset was investigated using Kaplan-Meier in the above subgroups of glioma patients. Results showed that the Met metabolism risk signature had a good predictive ability in IDH wt, IDH mutant-codel and IDH mutant-non-codel (*p* < 0.0001, *p* = 0.0068 and *p* = 0.025, respectively, Fig. S3A-C). To further examine the sensitivity and specificity of the Met metabolism risk signature in prognostic prediction in the above subgroups of glioma patients, time-dependent ROC curves were plotted. For IDH wt patients, the AUCs for 1-, 3-and 5-year OS were 0.68, 0.75 and 0.75, respectively (Fig. S3A). The AUCs for predicting 1-, 3- and 5-year OS of patients with IDH mutant-codel were 0.95, 0.66 and 0.86, respectively (Fig. S3B). The AUCs for predicting 1-, 3- and 5-year OS of patients with IDH mutant-non-codel were 0.73, 0.65 and 0.54, respectively (Fig. S3C). These results indicated that the model had a high accuracy in predicting overall survival of patients with gliomas.

### Validation of prognostic model by external datasets

**Fig. 3:**
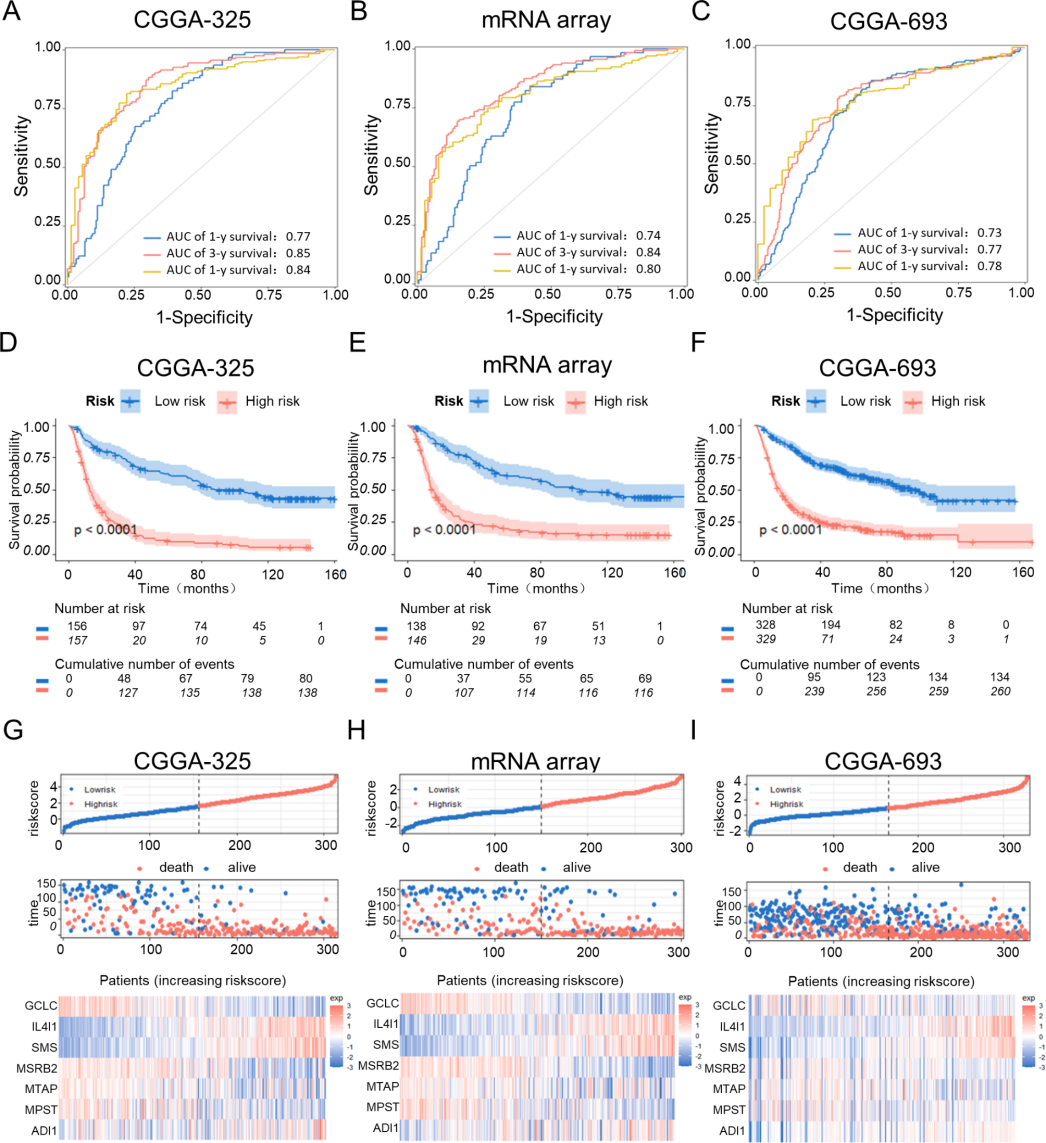
**Prognostic model evaluation and validation in validation sets. (A-C)** Time-dependent ROC curves for the signature of seven methionine metabolism genes in the cohorts (**A**) CGGA-325, (**B**) CGGA-mRNA array, and (**C**) CGGA-693. **(D–F)** KM curve of the prognosis signature in the **(D)** CGGA-325, **(E)** CGGA- mRNA array and **(F)** CGGA-693 cohorts (log‐rank test). **(G-I)** The riskscore, survival status and expression distribution of a signature based on seven methionine metabolism genes for glioma patients in the cohorts (**G**) CGGA-325, (**H**) CGGA-mRNA array, and (**I**) CGGA-693

To further evaluate the precision and dependability of the prognostic model, the predictive role of the risk score was verified using different glioma databases. In the CGGA325 database, the AUCs for 1-, 3-and 5-year OS were 0.77, 0.85 and 0.84, respectively (Fig. 3A). The AUCs for predicting 1-, 3- and 5-year OS in the CGGA (mRNA array) data set were 0.74, 0.84 and 0.80, respectively (Fig. 3B). The AUCs for predicting 1-, 3- and 5-year OS in the CGGA693 database data set were 0.73, 0.77 and 0.78, respectively (Fig. 3C). Additionally, the survival status and the expression distribution of the gene signature between the two risk groups were comparable to those in the TCGA database in these three external datasets (Fig. 3D-I). These results indicated that the prognostic model developed based on the Met metabolism genes profile had a great prediction value.

### Clinical and pathologic traits and the hallmark of the Met metabolism genes

**Fig. 4:**
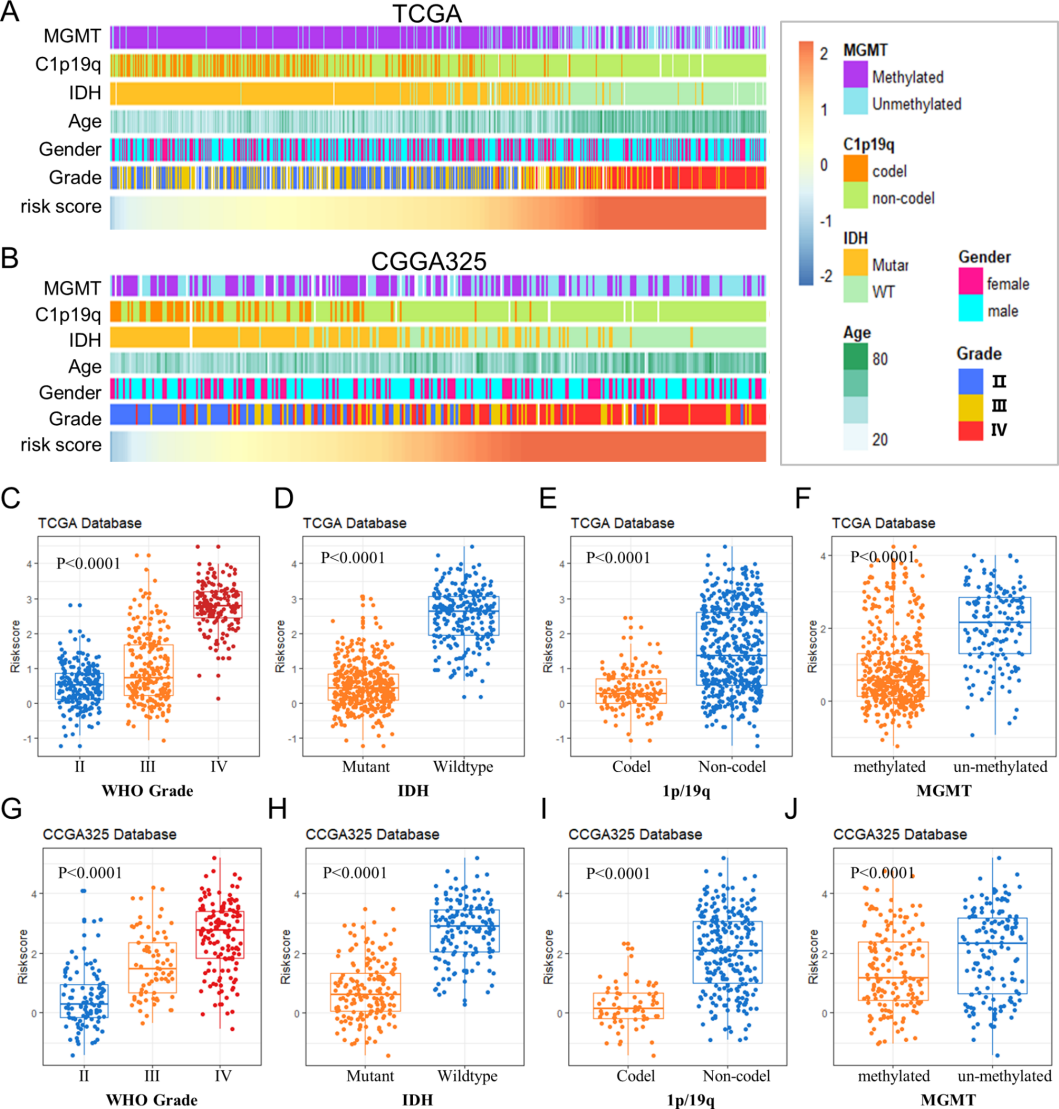
**The relationship between the Met metabolism genes signature and clinical characteristics. (A)** The landscape of Met metabolism gene signature-related clinic pathological features of gliomas in the TCGA database. **(B)** The landscape of Met metabolism gene signature‐related clinic pathological features of gliomas in the CGGA database. **(C-F)** The risk scores were significantly higher in gliomas with grade IV GBM, IDH wild-type, 1p/19q non-codeletion, or MGMT promoter unmethylation in the TCGA databases. **(G-J)** The risk scores were significantly higher in gliomas with grade IV GBM, IDH wild-type, 1p/19q non-codeletion, or MGMT promoter unmethylation in the CGGA databases; *P* < 0.0001; C,G:one-way ANOVA and Tukey’s test; D-F, H-J: unpaired t test. IDH, isocitrate dehydrogenase; WHO, world health organization

The relationship between the Met metabolism genes signature and clinical-pathologic factors was investigated based on the analysis of the risk scores from two independent RNA-seq databases. Complete 1p/19q co-deletion, MGMT promoter methylation and IDH1 mutation which are associated with favorable outcomes in gliomas were more obvious in the low-risk group as risk scores increased (all *p* < 0.001, Spearman correlation, Fig. 4A, B). In particular, IDH1 mutations were present in 92% of TCGA cases in the low-risk group (Supplementary Fig. S4), while in the TCGA and CGGA325 datasets there was a positive correlation between riskscores and age at diagnosis, WHO grades (Spearman correlation, Fig. 4A, B). Different groups of these samples were subjected to comparative analysis. GBM (grade IV) showed significantly higher risk scores in the TCGA and CGGA325 databases than glioma (grades II and III) (Fig. 4C, G). Furthermore, in both datasets, the samples with IDH-wildtype, 1p/19q non-codeletion, or MGMT promoter unmethylation had significantly higher risk scores (Fig. 4D-J). These findings showed a strong correlation between the malignant phenotype of gliomas and the Met metabolism gene profile.

### Functional annotation and chemotherapeutic sensitivity prediction of the Met metabolism gene signature

**Fig. 5:**
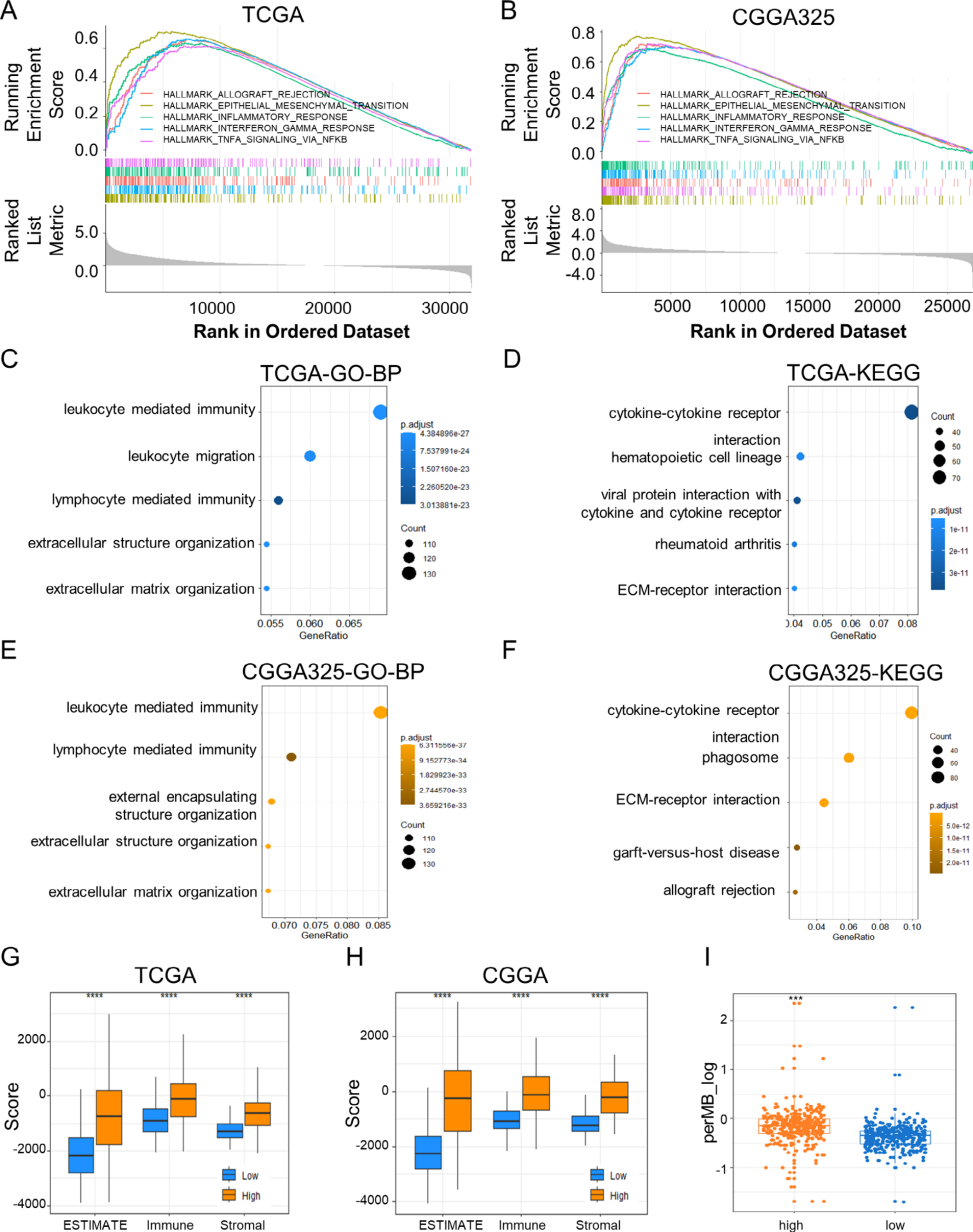
**Functional annotation of Met metabolism gene signature.** Gene set enrichment analysis (GSEA), Gene Ontology (GO) and Kyoto Encyclopedia of Genes and Genomes (KEGG) functional enrichment analysis based on the differential genes between high and low-risk groups in TCGA (**A, C, D**) and CGGA325 (**B, E, F**). The gene sets with the highest enrichment scores were displayed. GO-BP: GO biological process analysis. (**G, H**) ESTIMATE/immune/stromal scores were significantly up-regulated in the high-risk groups in the TCGA and CGGA databases. ***, *P* < 0.001; Wilcoxon test. (**I**) TMB (tumor mutation burden) increased in the high-risk group, ***, *P* < 0.001; Wilcoxon test

The biological functions and pathways associated with the risk score were explored through conducting differential gene analysis between high and low-risk groups, followed by functional enrichment and gene set enrichment (GSEA) analysis using the GO and KEGG. The PCA revealed transcriptomic differences between high- and low-risk groups in both databases, indicating that the two groups had distinct biological characteristics (Fig. S5A, B). The top gene sets (Hallmarker, GO biological processes, or KEGG) were associated with immune and inflammatory responses and extracellular matrix interactions, such as leukocyte mediated immunity, leukocyte migration, and epithelial mesenchymal transition (Fig. 5A-F). To further investigate the characteristics of the immune microenvironment in glioma of the high- and low‐risk groups, we performed ESTIMATE analysis to profile the immune characteristics in two glioma databases. The immune and stromal ESTIMATE scores were significantly higher in the high-risk group (Fig. 5G, H). Furthermore, we confirmed that the high-risk group had a higher tumor mutation burden (*p* < 0.001, Fig. 5I), indicating greater tumor heterogeneity and chemotherapy resistance. The ‘pRRophetic’ R package was then employed to predict the sensitivity of chemotherapeutic and targeted inhibitors in both databases for high- and low-risk groups. The top 12 compounds with the greatest negative correlations with the riskscore were selected (Fig. S5 C, D). Bortezomib, Cyclopamine, Docetaxel, and A-770,041 were among the top 12 in both databases, indicating a promising therapeutic activity for treating high-risk glioma patients.

### The development and validation of a nomogram for glioma prediction

**Fig. 6:**
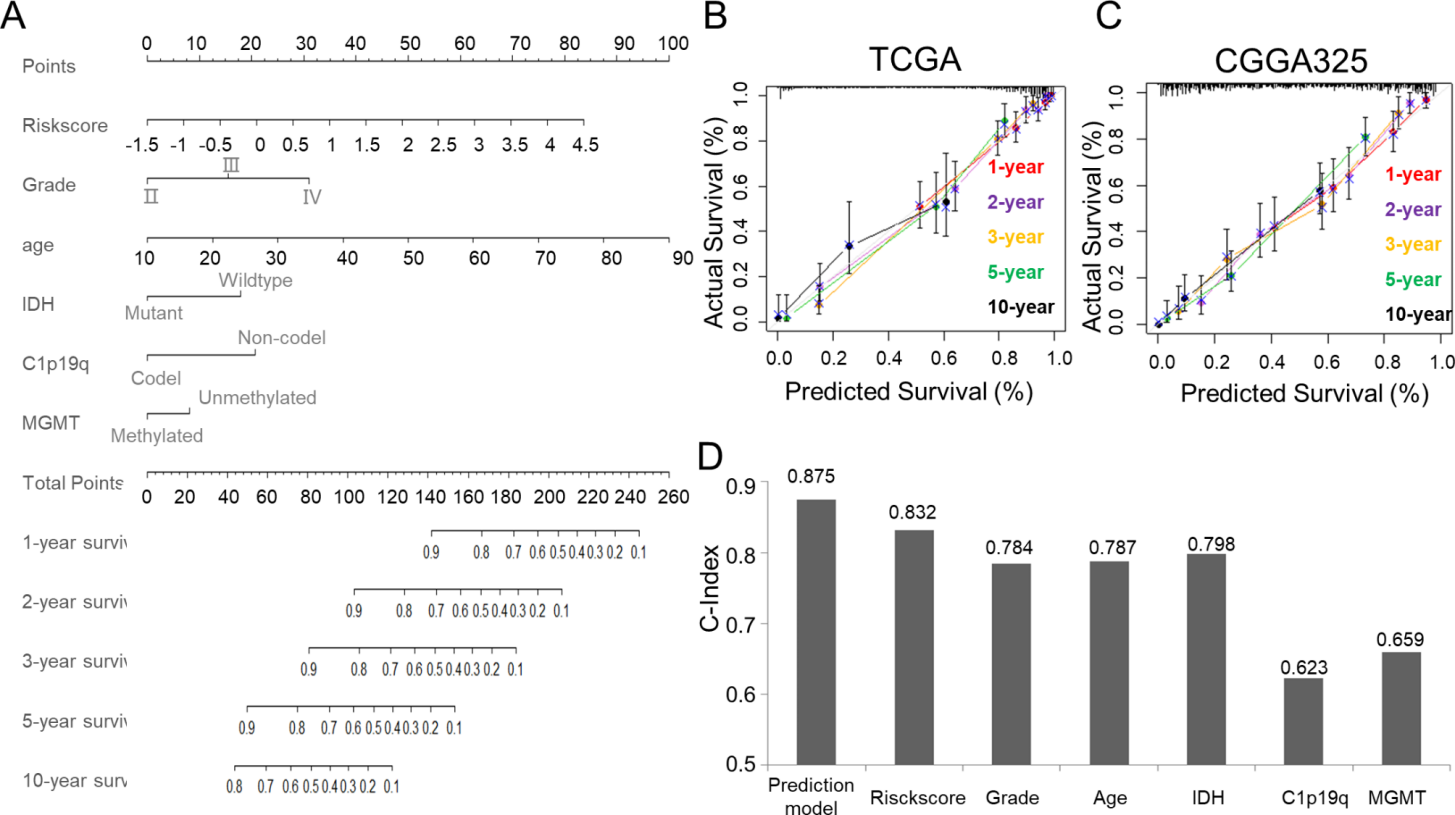
**The development and validation of a prediction nomogram for glioma** (**A**) Nomogram used independent prognostic factors to predict overall survival in glioma patients. (**B, C**) The calibration plots for predicting1-, 2-, 3-, 5- ,10- years OS in training and validation groups. (**D**) Comparison of C-index between prediction model, riskscore, and clinical prognostic factors used to predict overall survival

We then use multivariate Cox regression analysis to investigate whether the Met metabolism risk signature can predict prognosis independently. The Met metabolism risk signature was found to be a significant predictor of survival after adjusting for clinical factors that were significantly detrimental to survival in a univariable Cox model, including WHO grade, age at diagnosis, IDH status, 1p/19q status, and MGMT promoter status (Fig. S6). To further improve the clinical applicability of the prognostic prediction model, we developed a nomogram including the Met metabolism risk signature, WHO grade, age, IDH mutation status, 1p19q codeletion status, and MGMT promoter methylation status in the TCGA data set (Fig. 6A). In training and validation databases, the observed and optimism-corrected lines were well aligned in the calibration curve, indicating a high predictive accuracy (Fig. 6B, C). This nomogram model showed a C-index of 0.875, which was higher than the C-index of any other clinical prognostic factors, such as WHO grade, age, IDH mutation status, 1p19q codeletion status, and MGMT promoter methylation status, showing a high predictive accuracy (Fig. 6D).

## Discussion

Methionine addiction is a general hallmark of cancer [20]. Study found that methionine restriction inhibits glioma growth because gliomas have significantly higher methionine uptake and metabolic rate [21]. 11 C-methionine positron emission tomography is predictive of glioma prognosis [6]. However, comprehensive investigation into the predictive value of Met metabolism genes for glioma patients has not been conducted. The present study investigated the expression of Met metabolism genes in gliomas and their associations with OS. A prognostic model incorporating seven Met metabolism genes was developed and subsequently tested in external cohorts. Finally, a nomogram integrating the prognosis model with clinic pathological factors was developed for a convenient prediction of OS for glioma patients.

Previous studies have found that 11 C-methionine PET has a high sensitivity and specificity in indicating prognosis of glioma patients [5]. However, short half-life of 11 C (~ 20 min) is one of the main challenges of 11 C-methionine PET, as such a short half-life of 11 C limits 11 C-methionine PET studies to centers that could synthesize it. 18 F-FET PET with a longer half-life is used to assess gliomas in many countries [5]. The prognostic signature developed based on seven Met metabolism genes in this study performed well in predicting gliomas. The C-index of our Met metabolism risk signature was 0.83, which is higher than routine clinical examination items for glioma. The C-index [22–24] is a widely used metric for assessing the efficacy of a predictive or prognostic statistical model. The more closely a model’s C-index approaches 1, the more accurately it can classify results. IDH wt, IDH mutant-codeland samples with IDH mutant-non-codel have been proposed as classifications for glioma regardless of grade and histology. The Met metabolism risk signature was found to be highly predictive in IDH wt, IDH mutant-codel, and IDH mutant-non-codel subgroups of glioma patients. Furthermore, we established a nomogram for gliomas incorporating the Met metabolism prognostic signature and routine clinical examination items, with adequate discrimination and calibration. Nomograms are useful and accessible tools for physicians to predict survival, plan individualized treatment, and determine the interval of follow-up [25]. Hence, our model could facilitate methionine metabolism in the diagnosis of glioma and is highly promising in clinical promotion.

Methionine is an important amino acid that regulates glioma development and fuels a number of metabolic pathways. Our study discovered that the majority of Met metabolism genes were related to patients’ OS in the TCGA database (logrank p and Cox *p* < 0.05). These results indicated the potential role of Met metabolism genes in gliomas and the possibility of developing a prognostic model using these Met metabolism genes. The current Met metabolism prognostic model composed of seven genes (GCLC, IL4I1, SMS, MSRB2, MTAP, MPST, and ADI1), which could be classified into methionine de novo and salvage pathway (IL4I1, SMS, MTAP, and ADI1) and methionine and cysteine catabolism (GCLC, MSRB2, and MPST). IL4I1 encoding a secreted L-amino acid oxidase protein is mainly implicated in immune regulatory functions that have been attributed to the depletion of amino acids or the formation of H_2_O_2_ [26]. Moreover, IL4I1 could also promote glioma cell migration and metastasis [27]. SMS (spermine synthase) catalyzes the production of spermine from spermidine and is frequently upregulated in cancers, resulting in elevated polyamine level required for malignant transformation and tumor progression [28]. 5’-methylthioadenosine phosphorylase (MTAP) encodes a metabolic enzyme required for the metabolism of polyamines and purines, which leads to DNA synthesis. Homozygous deletion of MTAP in glioblastoma represents a potentially targetable vulnerability [29]. Although ADI1 (acireductone dioxygenase 1) as an enzyme in the methionine de novo and salvage pathway has been considered as a tumor suppressor in prostate cancer or hepatocellular carcinoma [30], our study found that higher ADI1 gene expression was associated with poor overall survival of glioma patients. Three additional genes involved in methionine and cysteine catabolism are linked to antioxidant activity. The mitochondrial protein methionine sulfoxide reductase B2 (MSRB2) protects cells against oxidative stress [31]. Similarly, the action of 3-mercaptopyruvate sulfurtransferase (MPST), a key enzyme that regulates endogenous H_2_S biosynthesis, is involved in protecting protein cysteine residues from harmful hyperoxidation [32]. GCLC (glutamate cysteine ligase) participates in the synthesis of glutathione from cysteine. High level of expression of these three genes was correlated with favorable overall survival. Antioxidants have been shown to be able to inhibit cancer initiation through promoting DNA repair and suppressing cancer progression [33]. On the other hand, some researches indicated that antioxidants could impair chemotherapeutic effects [34], in turn, this can also render the treatments less effective. In our study, GSS (glutathione synthetase) and GCLC had similar functions in synthesising glutathione, but they play opposite roles in promoting and inhibiting glioma progression. Therefore, more research is required to determine if antioxidants were harmful or beneficial for the treatment of gliomas.

Recent studies showed that Methionine could regulate the immune system [35]. Consistently, the Met metabolism genes signature was found to be associated with an enrichment of immune-related processes and pathways. Additionally, the signature of Met metabolism genes was typically correlated with TMB. Multiple studies have validated an association between TMB level and the efficacy of immunotherapy [36], suggesting that an effective Met metabolism risk signature could contribute to a better immunotherapy outcome. Nevertheless, gene mutation sites distribution showed that 92% of TCGA cases in low-risk group carried IDH1 mutation, suggesting a potential relationship between the Met metabolism risk signature and IDH mutation.

## Conclusions

In summary, our study developed a prognostic prediction model for glioma patients using seven Met metabolism genes. The model was closely associated with outcome status in both the derivation and validation cohorts, providing novel insights into the prediction of gliomas prognosis. Moreover, the prognostic Methionine metabolism genes were also related to the immune response and metastasis, which contributed to the development of more effective treatment strategies for glioma patients. Although this model has demonstrated the potential in improving understanding of methionine metabolism in gliomas and helps provide more optimal and promoting precise treatment for glioma patients, further validation in independent cohorts was equally important for clinical application.

### Electronic supplementary material

Below is the link to the electronic supplementary material.

Supplementary Material 1

## Data Availability

RNA-seq data were obtained from TCGA databases (https://tcgadata.nci.nih.gov) and CGGA databases (https://www.cgga.org.cn). Single-cell sequencing data were retrieved from the GSE11789112 data set of the Gene Expression Omnibus database.

## Abbreviations

Met C11: Methionine
CGGA: Chinese Glioma Genome Atlas
TCGA: The Cancer Genome Atlas
IDH: Isocitrate dehydrogenase
DEG: Differentially expressed gene
GO: Gene Ontology
KEGG: Kyoto Encyclopedia of Genes and Genomes
FDR: False discovery rate
LASSO: Least absolute shrinkage and selection operator
AIC: Akaike information criterion
