# Supplementary material for: Prognosis prediction based on methionine metabolism genes signature in gliomas
Source: BMC Med Genomics. 2023 Dec 6;16:317. doi: 10.1186/s12920-023-01754-x (PMC10699061; doi:10.1186/s12920-023-01754-x)
Supplement: Supplementary file 1 — Supplementary Material 1 [file 12920_2023_1754_MOESM1_ESM.pdf]

Supplementary Materials

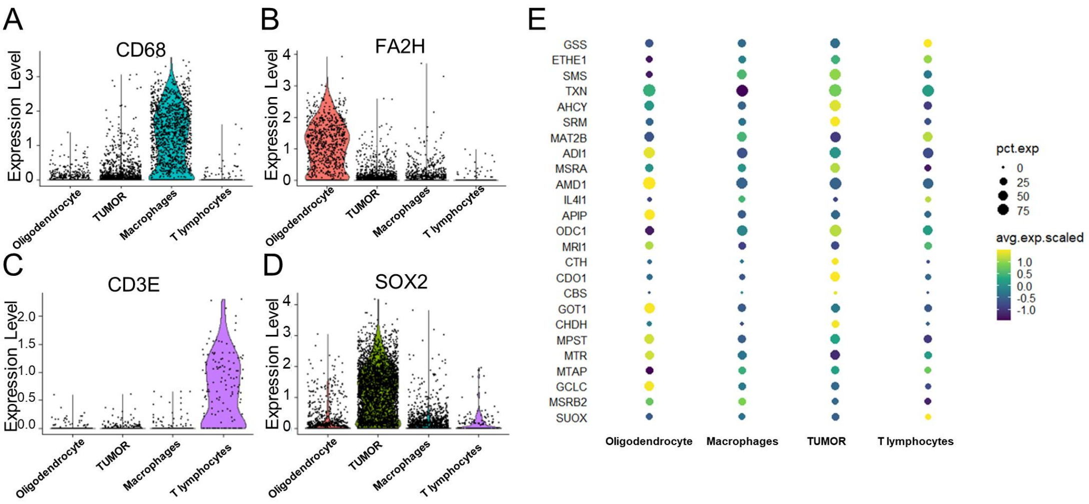

Fig.S1. Single-cell RNA-seq identifies Met metabolism genes. (A-D) CD68, FA2H, CD3E, and SOX2 are markers for macrophages, oligodendrocytes, T lymphocytes, and tumor cells, respectively. **(E)** Dotplot of the Met metabolism genes.

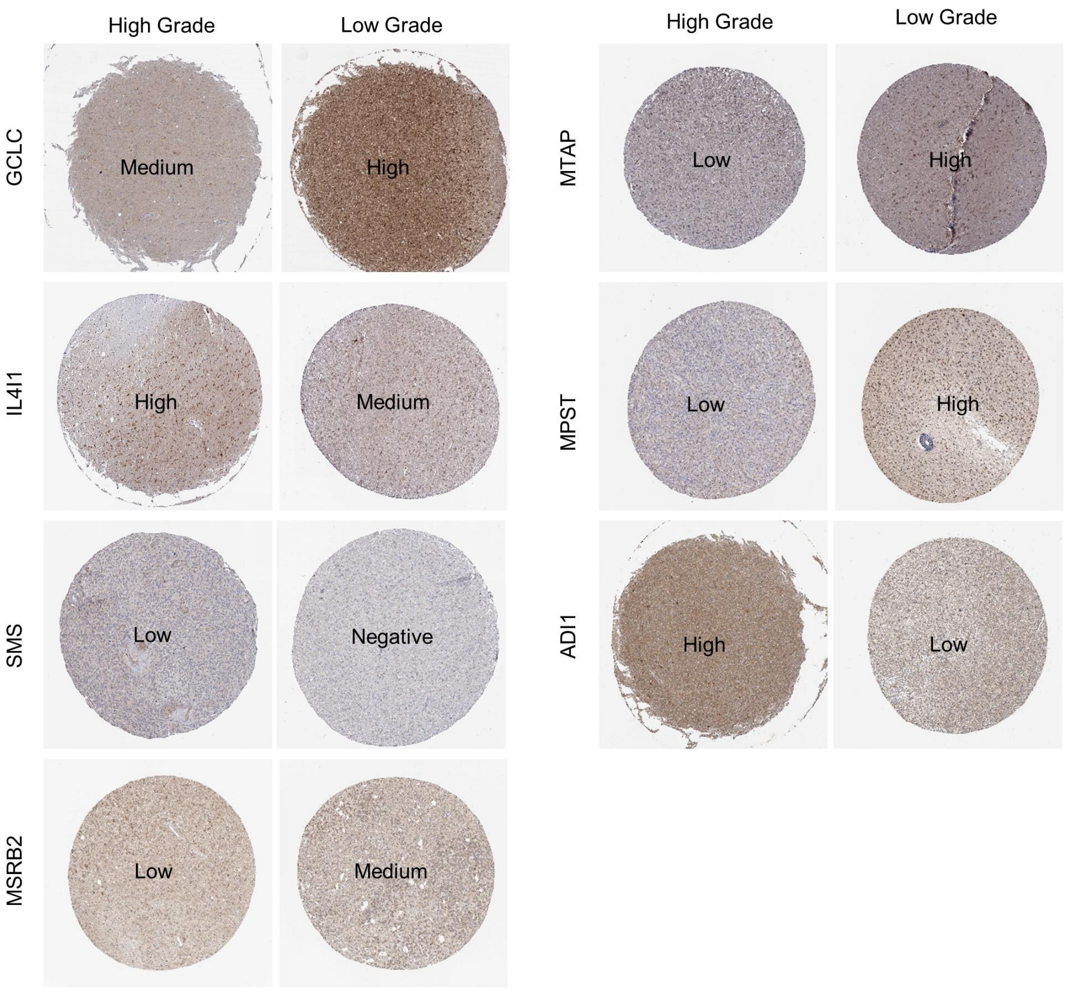

Fig.S2. The protein expression levels of the seven Met metabolism genes. The expression profiles of the proteins encoded by GCLC, IL4I1, SMS, MSRB2, MTAP, MPST, and ADI1 in low- and high-grade glioma tissues using clinical specimens from the Human Protein Profiles.

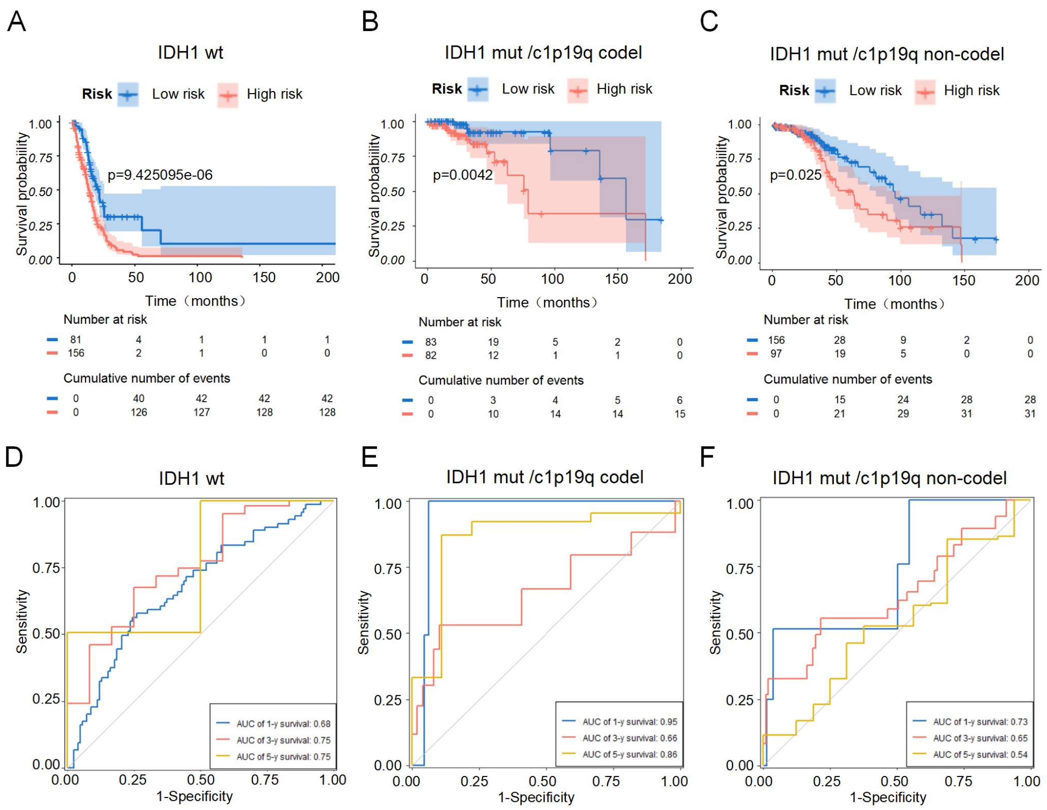

Fig. S3. Prognostic evaluation for the signature of seven methionine metabolism genes in the subgroups of glioma patients from TCGA database. (A-C) KM curve of the prognosis signature in the (**A**) IDH wild-type (IDH wt); (**B**) IDH mutations with chromosome 1p19q codeletions (IDH mutant-codel); (**C**) IDH mutations without chromosome 1p19q codeletions (IDH mutant-non-codel). The glioma patients in different subgroups were divided high risk group and low risk group according to the optimal cut-off value(minprop = 0.3). **(D-F)**Time-dependent ROC curves for the signature of seven methionine metabolism genes in the subgroups of glioma patients from TCGA database. (**D**)IDH wild-type (IDH wt); (**E**) IDH mutations with chromosome 1p19q codeletions (IDH mutant-codel); (**F**) IDH mutations without chromosome 1p19q codeletions (IDH mutant-non-codel).

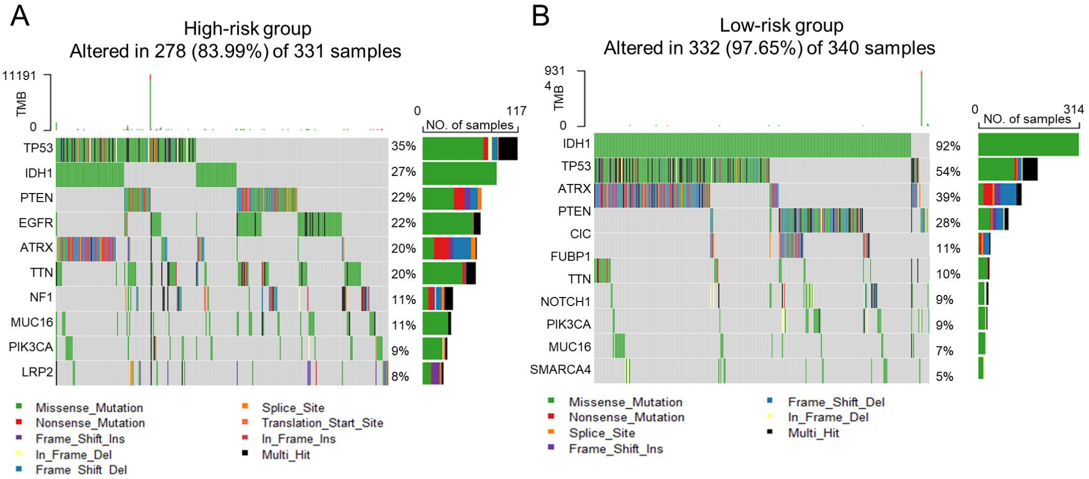

Fig.S4. TMB analysis in TCGA database. Gene mutation visualization for the high-risk (**A**) and low-risk groups (**B**).

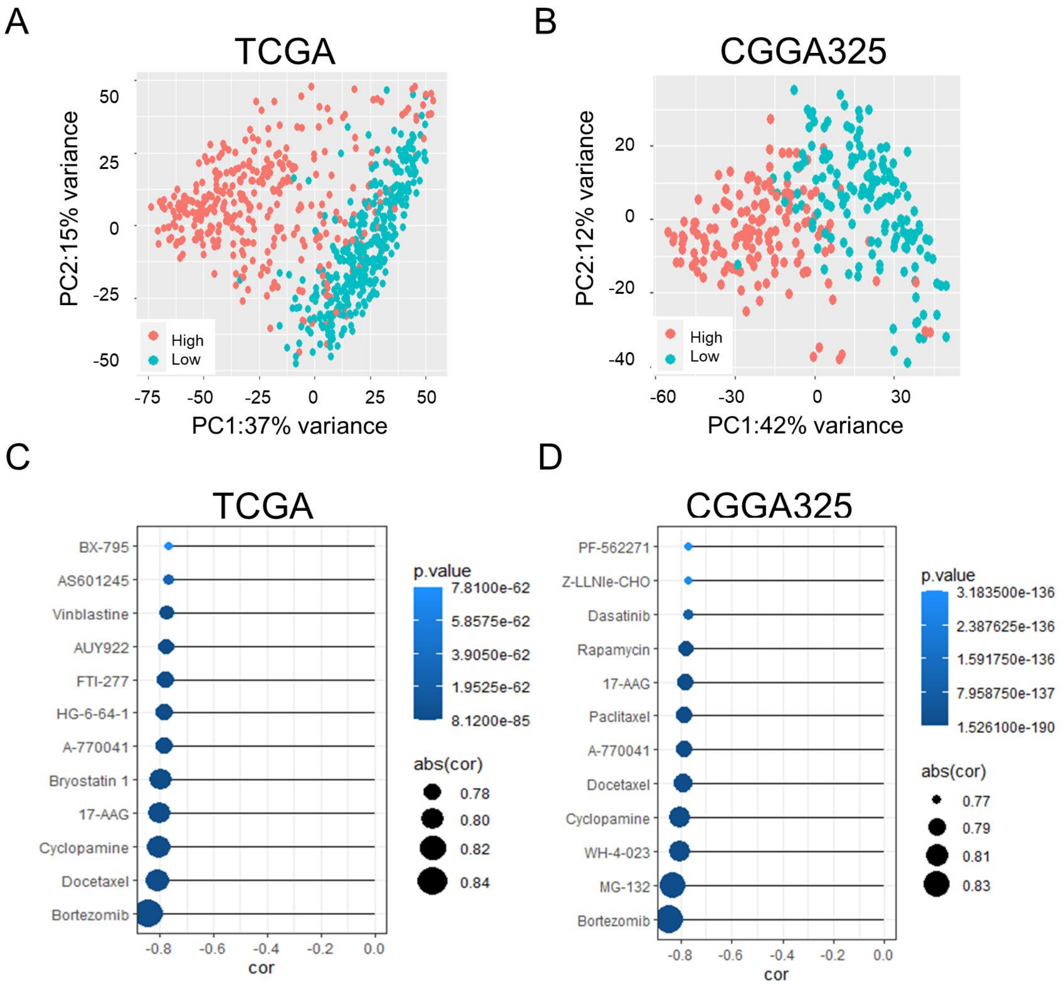

Fig.S5. PCA analysis and chemotherapeutic sensitivity prediction of chemotherapeutic of Met metabolism gene signature. (A, B) The PCA showed transcriptomic differences between high- and low-risk groups in both databases. **(C, D)** The top 12 compounds with the highest negative correlations with the riskscore in both databases.

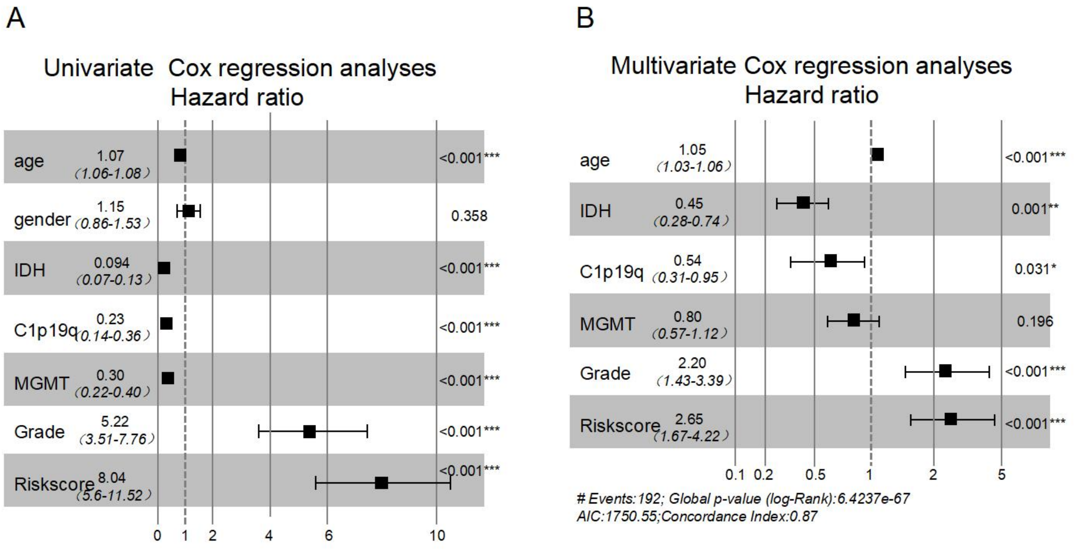

Fig.S6. Met metabolism risk signature is a prognostic factor in glioma patients. (A)Univariate Cox analyses of clinical prognostic parameters in TCGA databases. (B)Multivariate Cox analyses of clinical prognostic parameters in TCGA databases.
